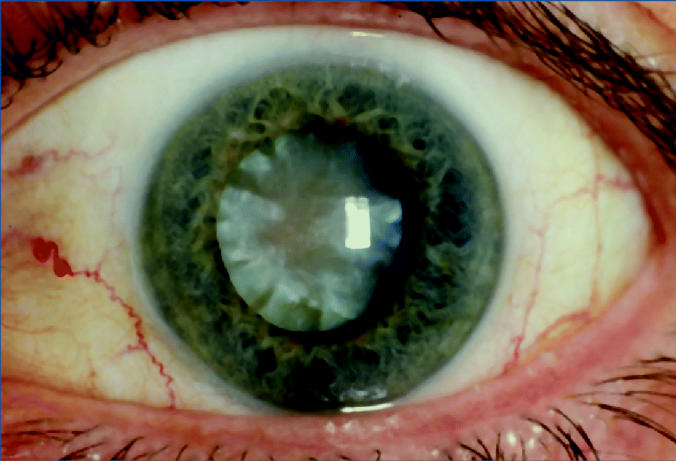# Headliners: Lead Exposure and Vision

**Published:** 2005-03

**Authors:** Jerry Phelps

## Lead Accumulation May Lead to Cataracts

Schaumberg DA, Mendes F, Balaram M, Dana MR, Sparrow D, Hu H. 2004. Accumulated lead exposure and risk of age-related cataract in men. JAMA 292:2750–2754.

Although lead toxicity in humans had been recognized for centuries, lead was widely used in industrial products and practices in the twentieth century, resulting in broad exposures and distribution of its effects. Worldwide, lead was a common component of many consumer products including gasoline, paint, craft supplies, and plumbing materials. Some of these routes of exposure still exist in countries outside the United States, making lead a lingering concern around the world.

Researchers have identified a number of adverse health effects of lead including neurotoxic effects and learning disorders in children. Other studies have shown that the intrusion of lead into the lens of the eye may cause protein conformational changes that decrease lens transparency. Now NIEHS grantee Howard Hu and colleagues at Harvard University have uncovered what could be another adverse health effect with global implications: cataracts.

Cataracts are the leading cause of blindness. About 13 million people over the age of 40 in the United States alone have cataracts, and the costs of cataract surgery reach almost $4 billion annually.

The Harvard researchers measured tibial and patellar bone lead levels by k X-ray fluorescence in a subset of participants in the Normative Aging Study, a Boston-based longitudinal study of aging in men. For 600 men aged 60 years and older, the researchers then reviewed eye examination data (collected routinely every 3–5 years) for the period after bone lead measurements were taken. Blood lead levels were also measured. Results were adjusted for pack-years of cigarette smoking, diabetes, blood lead, and intake of vitamin C, vitamin E, and carotenoids.

The researchers found that participants with high tibial lead were more than 2.5 times as likely to develop cataracts as men with low tibial lead (bone lead is a measure of long-term lead exposure). Blood lead levels, which are more indicative of short-term lead exposure, were not significantly associated with increased risk of cataract development.

This study suggests that accumulated lead exposure, common in the United States and other parts of the industrialized world, may be an important but as yet unrecognized risk factor for development of cataracts. Furthermore, reducing lead exposure could help decrease the global human suffering and financial burden caused by cataracts, and preserve the vision of many people as they age.

## Figures and Tables

**Figure:**